# Two Novel Nomograms Predicting the Risk and Prognosis of Pancreatic Cancer Patients With Lung Metastases: A Population-Based Study

**DOI:** 10.3389/fpubh.2022.884349

**Published:** 2022-05-31

**Authors:** Wei Zhang, Lichen Ji, Xugang Zhong, Senbo Zhu, Yi Zhang, Meng Ge, Yao Kang, Qing Bi

**Affiliations:** ^1^Department of Orthopedics, Zhejiang Provincial People's Hospital, Qingdao University, Qingdao, China; ^2^Department of Orthopedics, Zhejiang Provincial People's Hospital, Hangzhou, China; ^3^Department of Orthopedics, The Second Affiliated Hospital of Wenzhou Medical University, Wenzhou, China; ^4^Department of Hepatobiliary and Pancreatic Surgery and Minimally Invasive Surgery, Zhejiang Provincial People's Hospital, Affiliated People's Hospital, Hangzhou Medical College, Hangzhou, China; ^5^Graduate Department, Bengbu Medical College, Bengbu, China; ^6^Department of Orthopedics, Hangzhou Medical College People's Hospital, Hangzhou, China

**Keywords:** pancreatic cancer, lung metastasis, SEER database, predictive factors, overall survival, nomogram

## Abstract

**Background:**

Pancreatic cancer (PC) is one of the most common malignant types of cancer, with the lung being the frequent distant metastatic site. Currently, no population-based studies have been done on the risk and prognosis of pancreatic cancer with lung metastases (PCLM). As a result, we intend to create two novel nomograms to predict the risk and prognosis of PCLM.

**Methods:**

PC patients were selected from the Surveillance, Epidemiology, and End Results Program (SEER) database from 2010 to 2016. A multivariable logistic regression analysis was used to identify risk factors for PCLM at the time of diagnosis. The multivariate Cox regression analysis was carried out to assess PCLM patient's prognostic factors for overall survival (OS). Following that, we used area under curve (AUC), time-dependent receiver operating characteristics (ROC) curves, calibration plots, consistency index (C-index), time-dependent C-index, and decision curve analysis (DCA) to evaluate the effectiveness and accuracy of the two nomograms. Finally, we compared differences in survival outcomes using Kaplan-Meier curves.

**Results:**

A total of 803 (4.22%) out of 19,067 pathologically diagnosed PC patients with complete baseline information screened from SEER database had pulmonary metastasis at diagnosis. A multivariable logistic regression analysis revealed that age, histological subtype, primary site, N staging, surgery, radiotherapy, tumor size, bone metastasis, brain metastasis, and liver metastasis were risk factors for the occurrence of PCLM. According to multivariate Cox regression analysis, age, grade, tumor size, histological subtype, surgery, chemotherapy, liver metastasis, and bone metastasis were independent prognostic factors for PCLM patients' OS. Nomograms were constructed based on these factors to predict 6-, 12-, and 18-months OS of patients with PCLM. AUC, C-index, calibration curves, and DCA revealed that the two novel nomograms had good predictive power.

**Conclusion:**

We developed two reliable predictive models for clinical practice to assist clinicians in developing individualized treatment plans for patients.

## Introduction

Pancreatic cancer (PC) is one of the fatal cancers, accounting for 2.6% of newly diagnosed tumors and 4.7% of cancer-related deaths globally in 2020 (1). In a retrospective study of 13,233 patients with metastatic PC, 19.9% had lung metastasis, the second most common distant metastasis site beside liver metastasis (2). Once PC has metastasized, only 15–20% of tumors were resectable; even though 50–86% of these tumors were cured, there could be a local recurrence, resulting in a 5-year overall survival (OS) of only 10–20% (3). The median survival time for distant metastases in untreated patients does not exceed than 6 months (4). FOLFIRINOX and gemcitabine plus NAB-paclitaxel are the first-line regimens for treating metastatic PC. Patients' OS improved following effective systemic chemotherapy (5). Hence, distant metastases are a significant indicator of poor prognosis (6, 7).

At present, artificial intelligence has been widely used in various fields of public health. Mehbodniya et al. (8) used machine learning to classify fetal health from cardiotocographic data. Peng et al. (9) used an explainable artificial intelligence framework to predict deterioration risk of hepatitis patients. Hu et al. (10) used deep learning system to identify lymph node quantification and metastatic cancer. Nguyen et al. (11) used convolutional neural network to evaluate bone mineral density of hips based on Sobel gradient-based map of radiographs. Barbios et al. (12) used decision tree to guide performance of intraoperative liver biopsy during bariatric surgery.

Nomogram is a simple multivariable visualization tool used in oncology to predict and quantify individual patient survival to aid clinical decision-making and accurate prescription (13–16). Recently, a growing number of studies have reported using nomogram based on different demographic characteristics and clinicopathological data to predict the prognosis and risk of cancers such as esophageal, ovarian, and cervical cancers, contributing to the development of personalized oncology treatment (17–19). As a common site of distant metastasis of PC, lung metastases have devastating effects on the health of patients with PC. By analyzing the risk factors associated with lung metastases, we can make an early diagnosis of pancreatic cancer with lung metastases (PCLM). Accurately predicting OS allows physicians to better monitor patients. The prognostic factors of PCLM patient's OS are not clear, our purpose is to explore the prognostic factors affecting OS of PCLM patients, and to establish OS nomograms based on these factors. Revealing the prognostic factors of PCLM will help doctors formulate appropriate treatment plans, which is conducive to reducing the occurrence of lung-related events and improving the quality of patients' life.

However, the predictors of PCLM are not well-described, and most studies are limited to analyzing prognostic outcomes in small samples of single centers (4). The diagnosed and prognostic model for PCLM is still not well-constructed. Consequently, this study derived data from the Surveillance, Epidemiology, and End Results Program (SEER) database to in-depth analyze risk and prognostic factors affecting PCLM patients. More importantly, we were the first to develop predictive models for PCLM, and the model's results are realistic and feasible.

## Methods

### Data Source and Data Extraction

The data for this study were obtained from SEER database using SEER^*^Stat software version 8.3.5, including all newly diagnosed PC patients from 2010 to 2016. SEER database is a very authoritative database that collects tumor-related data of about 30% of the entire United States population, allowing us to draw a convincing conclusion (20). The SEER database completely records demographic characteristics, clinicopathological information and follow-up data of cancer patients. Because patient information in the SEER database is public and anonymous, ethical approval and patients' informed consent was not required for our study. Our research methods strictly adhere to the research standards published by SEER database.

**Figure 1:**
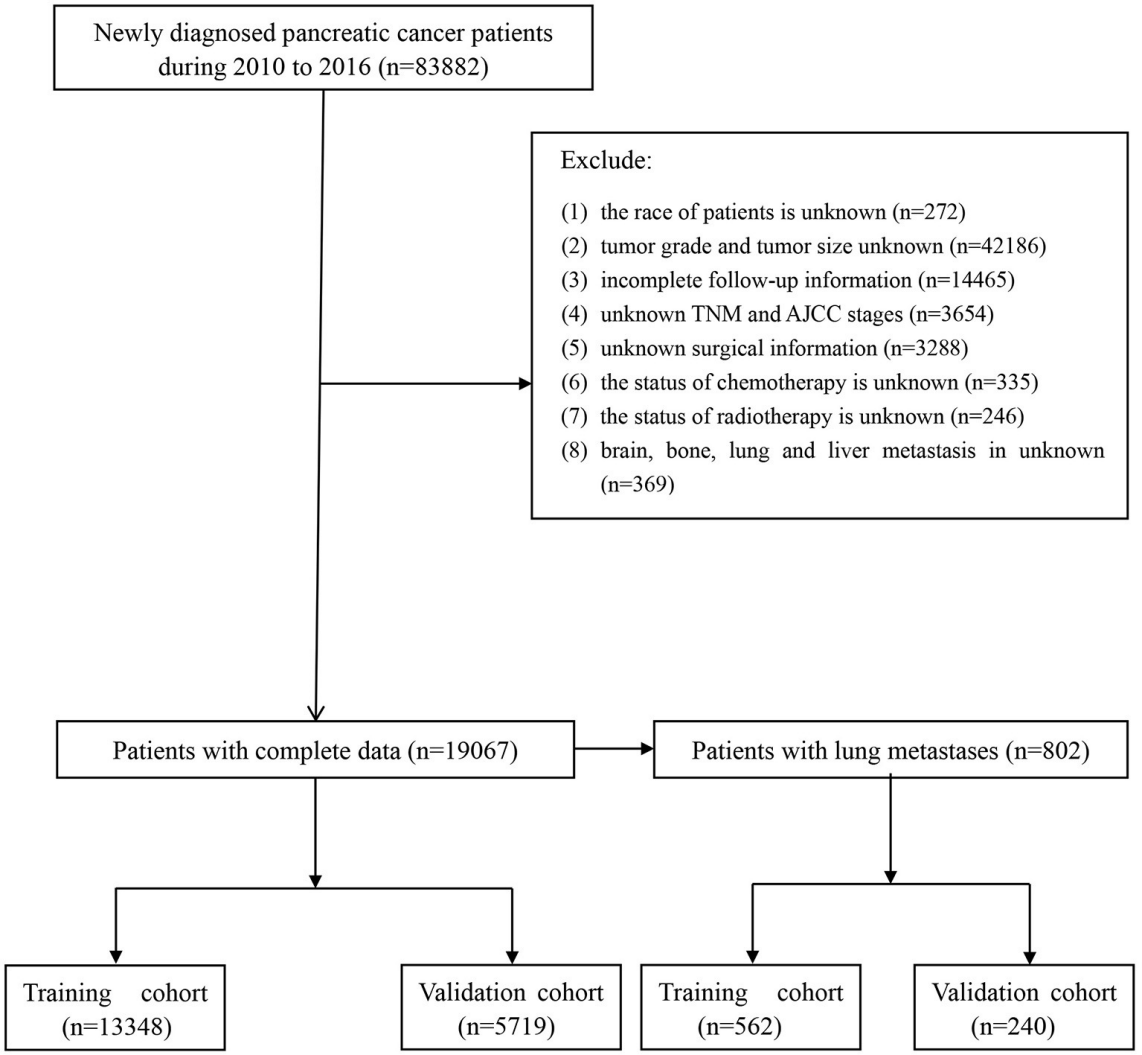
Flowchart of patients identified in this study.

The following continuous and categorical data were extracted conferring to the codes in the SEER database: age at diagnosis, race (white, black and other race), sex (female or male), histological subtype (adenocarcinoma, infiltrating duct carcinoma, neuroendocrine carcinoma and others), grade (well-differentiated, moderately differentiated, poorly differentiated and undifferentiated; anaplastic), primary site (head of the pancreas, body of the pancreas, the tail of pancreas, pancreatic duct, other specified parts of the pancreas, overlapping lesion of pancreas and pancreas NOS), T stage (T1, T2, T3, and T4), N stage (N0 and N1), therapy (surgery, radiotherapy or chemotherapy), tumor size, distant metastasis (brain metastasis, bone metastasis or liver metastasis). Inclusion criteria: (1) patients with a non-death certificate and non-autopsy confirmed diagnosis; (2) patients with complete survival and follow-up data; (3) patients with pancreatic cancer as the primary tumor; and (4) patients with a definite metastatic site, primary site, demographic characteristics, and histological information at the time of diagnosis. In this study, the primary outcome for prognostic survival was OS, defined as the time from diagnosis to the date of death or the last follow-up visit. The flowchart of patient screening is shown in Figure 1.

### Nomogram Construction and Validation

All patients were randomly divided into training and validation cohorts in a ratio of 7:3. In the training cohort, univariable and multivariable logistic regression models were used to analyze the independent risk factors of lung metastasis in patients with pancreatic cancer, and we established a nomogram to predict the risk of lung metastasis in PC patients. Then, univariate and multivariate Cox proportional hazard regression models were used to analyze the independent prognostic factors of patients with PCLM, and we also constructed nomograms to predict 6-, 12-, and 18-months OS of PCLM patients. We used area under curve (AUC), time-dependent receiver operating characteristics (ROC) curves, and calibration curves to verify the accuracy of training and validation cohort. In addition, we used consistency index (C-index) and time-dependent C-index to judge the discrimination ability of the model. Decision curve analysis (DCA) is a novel algorithm, which is often used to evaluate the clinical efficacy of the model.

### Statistical Analysis

Continuous variables were represented as medians and interquartile ranges (IQR), and categorical variables were as integers and percentages. The Mann-Whitney *U*-test was used to compare non-normally distributed continuous variables, and comparisons between categorical variables were assessed using the chi-square test or Fisher's exact test. On the initial cohort, we performed logistic regression analysis and incorporated variables from the univariable analysis with *P* < 0.05 into the multivariable analysis to obtain the odds ratio (OR) and corresponding 95% confidential interval (CI) for each independent risk factor. Cox proportional hazard regression analysis was then conducted on the training cohort. In multivariate Cox proportional hazard regression analysis, the variables with *P* < 0.1 in univariate analysis were included to obtain the hazard ratio (HR) and corresponding 95% CI for each independent prognostic variable. We developed two new nomograms based on these independent risk and prognostic factors. We plotted Kaplan-Meier curves to compare potential differences in OS among treatment methods, metastatic sites, grades, and histological subtypes. All statistical analyses were performed using SPSS 24.0 software (IBM, Chicago, IL, USA) and R software (version 4.0.2) (https://www.r-project.org/). A two-tailed *P* < 0.05 was considered statistically significant.

## Results

### Survival Outcomes

**Figure 2:**
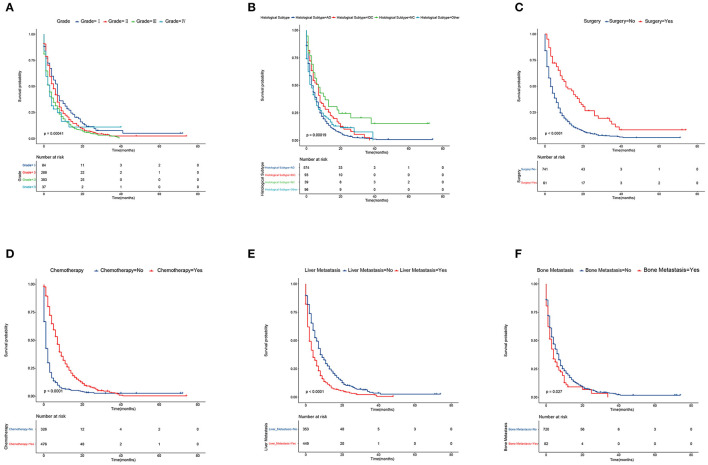
Kaplan–Meier analysis of overall survival among pancreatic cancer patients with lung metastasis at diagnosis. **(A)** grade, **(B)** histological subtype, **(C)** surgery, **(D)** chemotherapy, **(E)** liver metastasis, and **(F)** bone metastasis.

Survival outcomes obtained by the Kaplan-Meier method indicated that the median survival of patients with PCLM grade I was 6.50 months (IQR, 1.25–12.75 months); grade II, 4.50 months (IQR, 2.00–9.00 months); grade III, 2.00 months (IQR, 1.00–8.00 months); and grade IV, 2.00 months (IQR, 1.00–5.00 months) (Figure 2A). The median survival of patients with PCLM adenocarcinoma was 3.00 months (IQR, 1.00–8.00 months); Infiltrating duct carcinoma, 4.00 months (IQR, 2.00–10.50 months); Neuroendocrine carcinoma, 6.00 months (IQR, 3.00–18.00 months); and other, 2.50 months (IQR, 0.00–8.75 months) (Figure 2B). The median survival of patients with PCLM who underwent surgery was 10.00 months (IQR, 4.00–20.50 months); and did not underwent surgery, 3.00 months (IQR, 1.00–8.00 months) (Figure 2C). The median survival of patients with PCLM who received chemotherapy was 6.50 months (IQR, 3.00–11.00 months); and no chemotherapy, 1.00 months (IQR, 0.00–3.00 months) (Figure 2D). The median survival of patients with liver metastasis was 2.00 months (IQR, 1.00–6.00 months); and without liver metastasis, 5.00 months (IQR, 2.00–11.50 months) (Figure 2E). The median survival of patients with bone metastasis was 2.50 months (IQR, 1.00–6.00 months); without bone metastasis was 4.00 months (IQR, 1.00–8.00 months) (Figure 2F).

**Figure 3:**
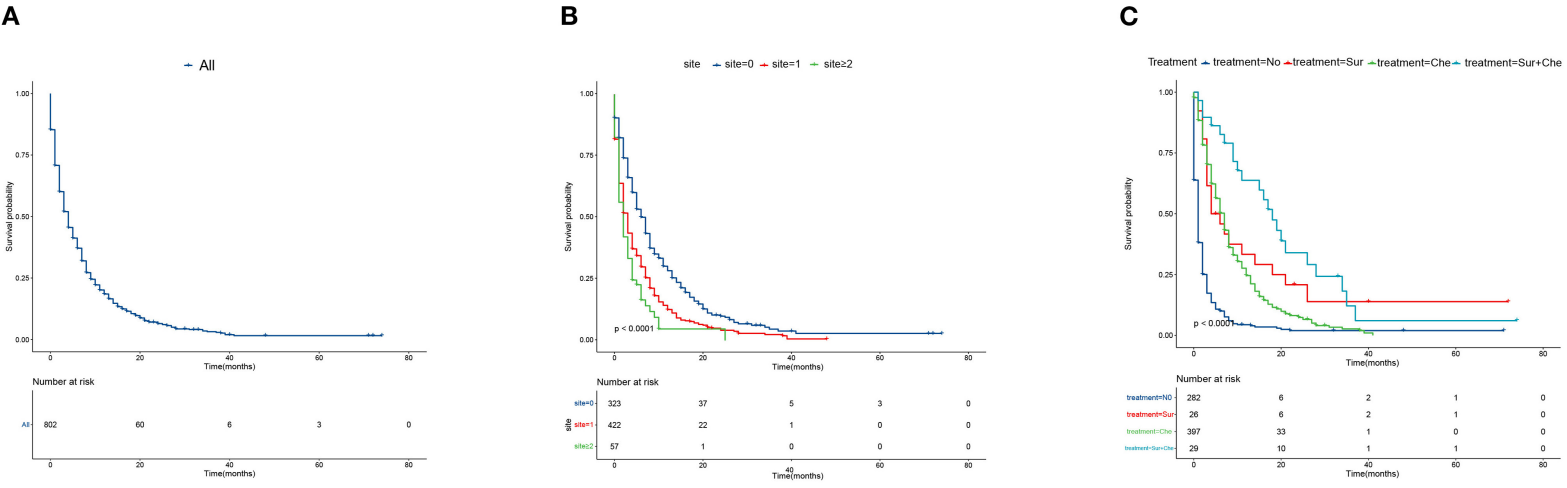
Kaplan–Meier analysis of overall survival among pancreatic cancer patients with lung metastasis at diagnosis. **(A)** overall, **(B)** stratified by the extent of extrapulmonary metastatic disease, and **(C)** stratified by type of treatment.

The median survival for all PCLM patients was 4.00 months (IQR, 1.00–8.00 months) (Figure 3A). We also established Kaplan-Meier curves of the number of metastatic sites and different treatments to compare the effects on survival outcomes. The median survival of those with no extrapulmonary metastatic site was 6.00 months (IQR, 2.00–12.00 months); 1 extrapulmonary metastatic site, 2.00 months (IQR, 1.00–7.00 months); with ≥2 extrapulmonary metastatic sites, 2.00 months (IQR, 1.00–4.00 months) (Figure 3B). The median survival months of patients with PCLM who received No treatment was 1.00 month (IQR, 0.00–2.00 months); Sur treatment, 4.50 months (IQR, 3.00–18.75 months); Che treatment, 6.00 months (IQR, 3.00–11.00 months); and Sur + Che treatment, 16.00 months (IQR, 7.00–23.50 months) (Figure 3C).

### Essential Characteristics of PCLM Patients

Complete data for all patients are shown in Table 1. There was no significant difference between the training cohort and the validation cohort. In the training cohort, a total of 13,348 PC patients between 2010 and 2016 met inclusion criteria, with 12,787 (95.8%) having no lung metastasis and 561 (4.2%) having lung metastases. 10,720 (80.3%) PC patients were white, and 6,428 (48.2%) were female. Adenocarcinoma was the most common histological subtype of pancreatic cancer, accounting for more than seven times as many cases as neuroendocrine carcinoma (51.3 vs. 7.6%). Furthermore, the head of the pancreas was the most common primary site of pancreatic cancer, contributing to 58.2%. In addition, the grade of PC patients was more concentrated in moderately differentiated (41.3%) and poorly differentiated (35.3%). According to the 7th AJCC stage, 7,719 (57.8%) were T3, and 9,822 (51.5%) were N1. In terms of therapy, 8,599 (64.4%) were treated with surgery, 10,642 (78.4%) did not receive radiotherapy, and 7,663 (57.4%) underwent chemotherapy. The median age of PCLM patients was 68 years (range: 60–76.5 years). PCLM patients were more likely to have poor differentiation when compared to those who did not have lung metastasis (G3: 48.1 vs. 34.7%, *p* < 0.001), no surgical treatment (92.2 vs. 33.1%, *p* < 0.001), liver metastasis (55.3 vs. 15.3%, *p* < 0.001), bone metastasis (10.2 vs. 0.9%, *p* < 0.001), and adenocarcinoma at the primary site (70.9 vs. 50.5%, *p* < 0.001). Patients' demographic characteristics and clinicopathological information are shown in Table 2.

**Table 1:** Demographic and clinicopathological characteristics in pancreatic cancer patients.

**Characteristic**	**Training cohort (*****n*** **=** **13,348)** **No. of patients %**		**Validation cohort (*****n*** **=** **5,719)** **No. of patients %**		***P-*value**
Age					0.417#
Median	67		67		
Range	59–75		59–74		
Race					0.552
White	10,720	80.3	4,556	79.7	
Black	1,470	11.0	658	11.5	
Other	1,158	8.7	505	8.8	
Sex					0.966
Female	6,428	48.2	2,756	48.2	
Male	6,920	51.8	2,963	51.8	
Histological subtype					0.298
Adenocarcinoma	6,854	51.3	2,966	51.9	
Infiltrating duct carcinoma	3,554	26.6	1,557	27.2	
Neuroendocrine carcinoma	1,008	7.6	426	7.4	
Other	1,932	14.5	770	13.5	
Grade					0.703
Well-differentiated: I	2,849	21.3	1,210	21.2	
Moderately differentiated: II	5,509	41.3	2,335	40.8	
Poorly differentiated: III	4,708	35.3	2,040	35.7	
Undifferentiated; anaplastic: IV	282	2.1	134	2.3	
Primary site					0.309
Head of pancreas	7,766	58.2	3,399	59.4	
Body of pancreas	1,625	12.2	680	11.9	
Tail of pancreas	2,234	16.7	919	16.1	
Pancreatic duct	65	0.5	19	0.3	
Other specified parts of pancreas	227	1.7	80	1.4	
Overlapping lesion of pancreas	868	6.5	371	6.5	
Pancreas, NOS	563	4.2	251	4.4	
AJCC T stage					0.520
T1	1,264	9.5	508	8.9	
T2	2,592	19.4	1,136	19.9	
T3	7,719	57.8	3,296	57.6	
T4	1,773	13.3	779	13.6	
AJCC N stage					0.900
N0	6,476	48.5	2,769	48.4	
N1	6,872	51.5	2,950	51.6	
Surgery					0.552
No	4,749	35.6	2,009	35.1	
Yes	8,599	64.6	3,710	64.9	
Radiotherapy					0.564
No	10,462	78.4	4,461	78.0	
Yes	2,886	21.6	1,258	22.0	
Chemotherapy					0.256
No	5,685	42.6	2,385	41.7	
Yes	7,663	57.4	3,334	58.3	
Tumor size					0.462#
Median	35		35		
Range	25–46		25–46		
Brain metastasis					0.536
No	13,332	99.9	5,714	99.9	
Yes	16	0.1	5	0.1	
Bone metastasis					0.831
No	13,182	98.8	5,650	98.8	
Yes	166	0.2	69	0.2	
Liver metastasis					0.704
No	11,082	83.0	4,761	83.2	
Yes	2,266	17.0	958	16.8	
Lung metastasis					0.841
No	12,787	95.8	5,475	95.7	
Yes	561	4.2	244	4.3	

*# Mann-Whitney U-test*.

**Table 2:** Demographic and clinicopathological characteristics of pancreatic cancer patients with lung metastases in training cohort.

**Characteristic**	**Without LM cohort (*****n*** **=** **12,787)** **No. of patients %**		**With LM cohort (*****n*** **=** **561)** **No. of patients %**		***P-*value**
Age					0.013#
Median	67		68		
Range	59–75		60–76.5		
Race					0.125
White	10,286	80.4	434	77.4	
Black	1,394	10.9	76	13.5	
Other	1,107	8.7	51	9.1	
Sex					0.087
Female	6,138	48.0	290	51.7	
Male	6,649	52.0	271	48.3	
Histological subtype					<0.001
Adenocarcinoma	6,456	50.5	398	70.9	
Infiltrating duct carcinoma	3,484	27.2	70	12.5	
Neuroendocrine carcinoma	981	7.7	27	4.8	
Other	1,866	14.6	66	11.8	
Grade					<0.001
Well-differentiated: I	2,794	21.9	55	9.8	
Moderately differentiated: II	5,299	41.4	210	37.4	
Poorly differentiated: III	4,438	34.7	270	48.1	
Undifferentiated; anaplastic: IV	256	2.0	26	4.6	
Primary site					<0.001
Head of pancreas	7,551	59.1	215	38.3	
Body of pancreas	1,518	11.9	107	19.1	
Tail of pancreas	2,098	16.4	136	24.2	
Pancreatic duct	61	0.5	4	0.7	
Other specified parts of pancreas	218	1.7	9	1.6	
Overlapping lesion of pancreas	814	6.4	54	9.6	
Pancreas, NOS	527	4.1	36	6.4	
AJCC T stage					<0.001
T1	1,246	9.7	18	3.2	
T2	2,418	18.9	174	31.0	
T3	7,511	58.7	208	37.1	
T4	1,612	12.6	161	28.7	
AJCC N stage					0.007
N0	6,235	48.8	241	43.0	
N1	6,552	51.2	320	57.0	
Surgery					<0.001
No	4,232	33.1	517	92.2	
Yes	8,555	66.9	44	7.8	
Radiotherapy					<0.001
No	9,952	77.8	510	90.9	
Yes	2,835	22.2	51	9.1	
Chemotherapy					0.340
No	5,457	42.7	228	40.6	
Yes	7,330	57.3	333	59.4	
Tumor size					<0.001#
Median	35		45		
Range	25–45		33–60		
Brain metastasis					<0.001
No	12,779	99.9	553	98.6	
Yes	8	0.1	8	1.4	
Bone metastasis					<0.001
No	12,678	99.1	504	89.8	
Yes	109	0.9	57	10.2	
Liver metastasis					<0.001
No	10,831	84.7	251	44.7	
Yes	1,956	15.3	310	55.3	

*# Mann-Whitney U-test*.

### Risk Factors for Developing Lung Metastasis in SEER Cohort

First, we carefully analyzed the risk factors significantly associated with pancreatic cancer developing lung metastasis. Univariable and multivariable logistic regression results are shown in Table 3. The variables with *p* < 0.05 in univariable logistic regression were then included in multivariable logistic regression analysis, age at diagnosed, histological subtype, primary site, N stage, surgery, radiotherapy, tumor size, brain metastasis, bone metastasis and liver metastasis were finally determined to be independent risk factors.

**Table 3:** Univariable and multivariable logistic regression of risk factor of lung metastasis in pancreatic carcinoma patients.

**Characteristics**	**Univariate analysis**		**Multivariate analysis**	
	**OR (95% CI)**	***P***	**OR (95% CI)**	***P***
Age	1.009 (1.002–1.017)	0.017	1.008 (1.001–1.015)	0.026
**Race**				
White	Reference			
Black	1.292 (0.999–1.649)	0.045		
Other	1.092 (0.802–1.454)	0.561		
**Sex**				
Female	Reference			
Male	0.863 (0.728–1.022)	0.087		
**Histological subtype**				
Adenocarcinoma	Reference		Reference	
Infiltrating duct carcinoma	0.326 (0.250–0.419)	<0.001	1.599 (1.180–2.143)	0.002
Neuroendocrine carcinoma	0.446 (0.294–0.650)	<0.001	0.661 (0.416–1.013)	0.068
Other	0.574 (0.436–0.743)	<0.001	0.757 (0.556–1.016)	0.070
**Grade**				
Well-differentiated: I	Reference			
Moderately differentiated: II	2.013 (1.503–2.743)	<0.001		
Poorly differentiated: III	3.091 (2.323–4.186)	<0.001		
Undifferentiated; anaplastic: IV	5.159 (3.136–8.286)	<0.001		
**Primary site**				
Head of pancreas	Reference		Reference	
Body of pancreas	2.476 (1.945–3.134)	<0.001	1.664 (1.283–2.149)	<0.001
Tail of pancreas	2.277 (1.823–2.833)	<0.001	2.072 (1.608–2.663)	<0.001
Pancreatic duct	2.303 (0.695–5.651)	0.109	2.210 (0.602–6.388)	0.416
Other specified parts of pancreas	1.450 (0.682–2.701)	0.284	0.916 (0.418–1.778)	0.559
Overlapping lesion of pancreas	2.330 (1.699–3.143)	<0.001	1.172 (0.831–1.631)	0.048
Pancreas, NOS	2.399 (1.643–3.408)	<0.001	1.436 (0.958–2.099)	0.072
**AJCC T stage**				
T1	Reference			
T2	4.981 (3.140–8.416)	<0.001		
T3	1.917 (1.215–3.225)	0.008		
T4	6.914 (4.345–11.707)	<0.001		
**AJCC N stage**				
N0	Reference		Reference	
N1	1.264 (1.066–1.500)	0.007	2.124 (1.754–2.575)	<0.001
**Surgery**				
No	Reference		Reference	
Yes	0.042 (0.030–0.057)	<0.001	0.051 (0.035–0.073)	<0.001
**Radiotherapy**				
No	Reference		Reference	
Yes	0.351 (0.259–0.465)	<0.001	0.503 (0.360–0.687)	<0.001
**Chemotherapy**				
No	Reference			
Yes	1.087 (0.916–1.293)	0.340		
Tumor size	1.021 (1.018–1.024)	<0.001	1.008 (1.003–1.012)	<0.001
**Brain metastasis**				
No	Reference		Reference	
Yes	23.108 (8.474–63.021)	<0.001	7.883 (2.560–24.038)	<0.001
**Bone metastasis**				
No	Reference		Reference	
Yes	13.154 (9.381–18.265)	<0.001	4.035 (2.775–5.820)	<0.001
**Liver metastasis**				
No	Reference		Reference	
Yes	6.839 (5.753–8.137)	<0.001	1.652 (1.352–2.021)	<0.001

### Construction and Validation of a Diagnostic Nomogram

**Figure 4:**
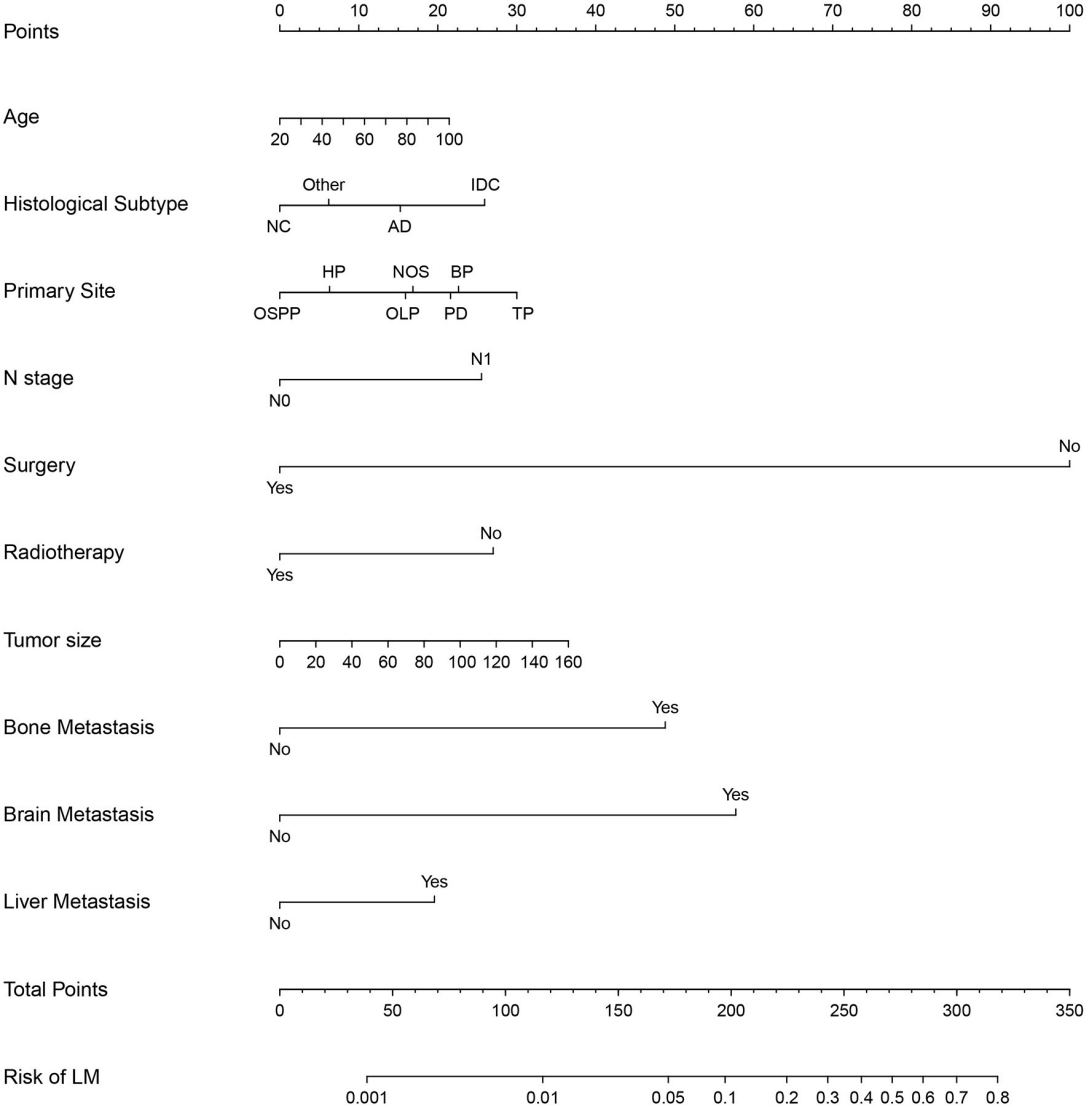
Nomogram to estimate the risk factors of lung metastasis in patients with pancreatic cancer. HP, head of pancreas; BP, body of pancreas; OLP, overlapping lesion of pancreas; OSPP, other specified parts of pancreas; TP, tail of pancreas; PD, pancreatic duct; AC, Adenocarcinoma; IDC, Infiltrating duct carcinoma; NC, neuroendocrine carcinoma.

**Figure 5:**
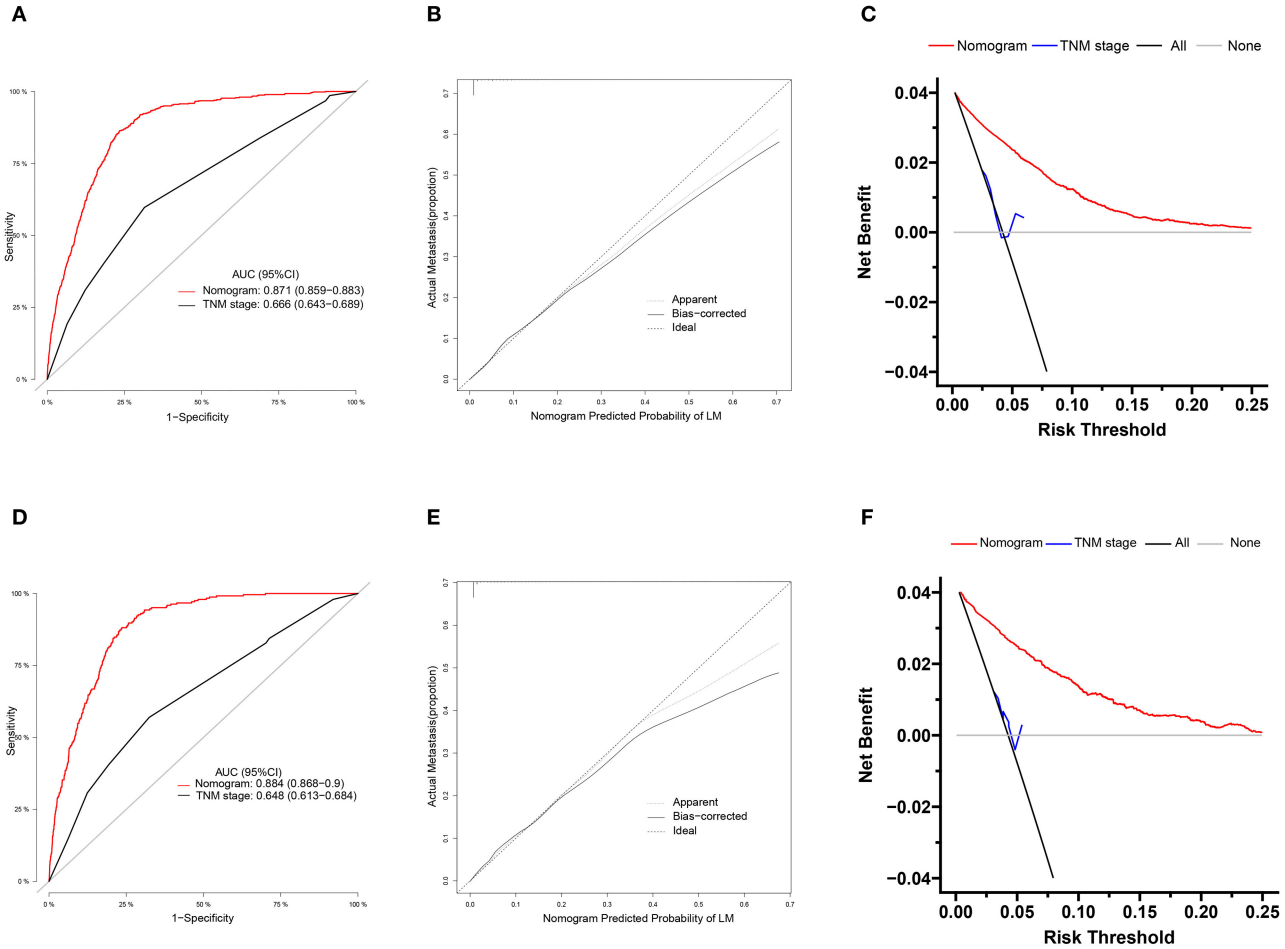
ROC curves, calibration plots and decision curves of the nomogram for the risk of pancreatic cancer with lung metastasis. **(A)** The AUC was utilized to judge the advantages and disadvantages of nomogram. **(B)** Calibration plot for the diagnostic nomogram. The diagonal 45-degree line indicates perfect prediction. **(C)** Decision curve analysis for the diagnostic nomogram. The net benefit calculated by adding true positive and minus the false positive corresponds to the measurement of Y-axis; X-axis represents the threshold probability. **(D–F)** The AUC, calibration plot and decision curve in validation cohort.

We established a predictive diagnostic nomogram based on the independent risk factors identified through multivariable logistic regression analysis (Figure 4). We created an easier-to-use free browser-based online calculator available at https://pclmnomogram.shinyapps.io/DynNomapp/. Many methods, including the AUC, calibration curves, and DCA, were used to assess the nomogram's differential advantage. As compared to the traditional TNM stage, the AUC value of the nomogram reflected better accuracy of the model (0.871, 95% CI: 0.859–0.883 vs. 0.666, 95% CI: 0.643–0.689 and 0.884, 95% CI: 0.868–0.900 vs. 0.648, 95% CI: 0.613–0.684) (Figures 5A,D). The calibration curves illustrated that model prediction was in good agreement with actual observation (Figures 5B,E). DCA displayed net benefits of the nomogram and traditional TNM staging both in training cohort and validation cohort (Figures 5C,F).

### Prognostic Factors of PCLM

Table 4 describes the baseline characteristics and clinical information of PCLM patients in depth. There was no significant difference between the training set and the validation set. As demonstrated in Table 5, The variables with *p* < 0.1 in univariate analysis were included in multivariate hazard Cox regression analysis. We finally eliminated eight statistically significant independent prognostic factors, including age at diagnosis, histological subtype, grade, surgery, chemotherapy, tumor size, bone metastasis, and liver metastasis.

**Table 4:** Demographic and clinicopathological characteristics in pancreatic cancer patients with lung metastasis.

**Characteristic**	**Training cohort (*****n*** **=** **562)** **No. of patients %**		**Validation cohort (*****n*** **=** **240)** **No. of patients %**		***P-*value**
Age					0.031#
Median	68		70		
Range	60–76		63–77		
Race					0.616
White	428	76.2	190	79.2	
Black	74	13.2	29	12.1	
Other	60	10.6	21	8.7	
Sex					0.028
Female	271	48.2	136	56.7	
Male	291	51.8	104	43.3	
Histological subtype					0.367
Adenocarcinoma	402	71.5	172	71.7	
Infiltrating duct carcinoma	63	11.2	30	12.5	
Neuroendocrine carcinoma	32	5.7	7	2.9	
Other	65	11.6	31	12.9	
Grade					0.768
Well-differentiated: I	61	10.9	23	9.6	
Moderately differentiated: II	198	35.2	90	37.5	
Poorly differentiated: III	279	49.6	114	47.5	
Undifferentiated; anaplastic: IV	24	4.3	13	5.4	
Primary site					0.600
Head of pancreas	215	38.3	97	40.4	
Body of pancreas	102	18.1	51	21.3	
Tail of pancreas	143	25.4	46	19.2	
Pancreatic duct	4	0.7	0	0	
Other specified parts of pancreas	5	0.9	6	2.5	
Overlapping lesion of pancreas	52	9.3	30	12.5	
Pancreas, NOS	41	7.3	10	4.2	
AJCC T stage					0.265
T1	16	2.8	11	4.6	
T2	173	30.8	78	23.5	
T3	223	39.7	80	33.3	
T4	150	26.7	71	29.6	
AJCC N stage					0.920
N0	241	42.9	102	42.5	
N1	321	57.1	138	57.5	
Surgery					0.512
No	517	92.0	224	93.3	
Yes	45	8.0	16	6.7	
Radiotherapy					0.923
No	514	91.5	220	91.7	
Yes	48	8.5	20	8.3	
Chemotherapy					0.821
No	227	40.4	99	41.3	
Yes	335	59.6	141	58.7	
Tumor size					0.257#
Median	45		44		
Range	34–60		32–58		
Brain metastasis					0.186
No	558	99.3	235	97.9	
Yes	4	0.7	5	2.1	
Bone metastasis					0.159
No	499	88.8	221	92.1	
Yes	63	11.2	19	7.9	
Liver metastasis					0.498
No	243	43.2	110	45.8	
Yes	319	56.8	130	54.2	

*# Mann-Whitney U-test*.

**Table 5:** Univariate and multivariate Cox regression of prognostic factors of lung metastasis in pancreatic carcinoma patients.

**Characteristics**	**Univariate analysis**		**Multivariate analysis**	
	**HR (95% CI)**	***P***	**HR (95% CI)**	***P***
Age	1.016 (1.008–1.024)	<0.001	1.014 (1.005–1.023)	0.002
**Race**				
White	Reference			
Black	1.223 (0.940–1.590)	0.133		
Other	1.130 (0.846–1.511)	0.408		
**Sex**				
Female	Reference			
Male	1.232 (1.034–1.468)	0.019		
**Histological subtype**				
Adenocarcinoma	Reference		Reference	
Infiltrating duct carcinoma	0.721 (0.541–0.960)	0.025	0.929 (0.687–1.258)	0.635
Neuroendocrine carcinoma	0.496 (0.330–0.745)	<0.001	0.385 (0.251–0.591)	<0.001
Other	0.998 (0.756–1.317)	0.989	0.905 (0.671–1.220)	0.513
**Grade**				
Well-differentiated: I	Reference		Reference	
Moderately differentiated: II	1.256 (0.920–1.715)	0.151	1.167 (0.840–1.621)	0.359
Poorly differentiated: III	1.731 (1.282–2.335)	<0.001	1.649 (1.191–2.285)	0.003
Undifferentiated; anaplastic: IV	1.992 (1.172–3.384)	0.011	1.506 (0.864–2.624)	0.148
**Primary site**				
Head of pancreas	Reference			
Body of pancreas	1.115 (0.867–1.433)	0.397		
Tail of pancreas	1.396 (1.116–1.744)	0.003		
Pancreatic duct	1.326 (0.492–3.572)	0.577		
Other specified parts of pancreas	1.318 (0.542–3.209)	0.542		
Overlapping lesion of pancreas	1.280 (0.932–1.759)	0.127		
Pancreas, NOS	1.169 (0.823–1.659)	0.383		
**AJCC T stage**				
T1	Reference			
T2	1.873 (1.097–3.197)	0.021		
T3	1.424 (0.840–2.413)	0.189		
T4	1.980 (1.155–3.393)	0.013		
**AJCC N stage**				
N0	Reference			
N1	1.067 (0.894–1.272)	0.474		
**Surgery**				
No	Reference		Reference	
Yes	0.446 (0.318–0.626)	<0.001	0.487 (0.334–0.710)	<0.001
**Radiotherapy**				
No	Reference			
Yes	0.911 (0.668–1.242)	0.555		
**Chemotherapy**				
No	Reference		Reference	
Yes	0.373 (0.311–0.447)	<0.001	0.291 (0.239–0.355)	<0.001
Tumor size	1.008 (1.004–1.012)	<0.001	1.007 (1.002–1.012)	0.004
**Brain metastasis**				
No	Reference			
Yes	1.523 (0.569–4.079)	0.402		
**Bone metastasis**				
No	Reference		Reference	
Yes	1.280 (0.967–1.695)	0.084	1.737 (1.290–2.339)	<0.001
**Liver metastasis**				
No	Reference		Reference	
Yes	1.657 (1.386–1.980)	<0.001	1.321 (1.088–1.605)	0.005

### Prognostic Nomograms Establishment and Validation

**Figure 6:**
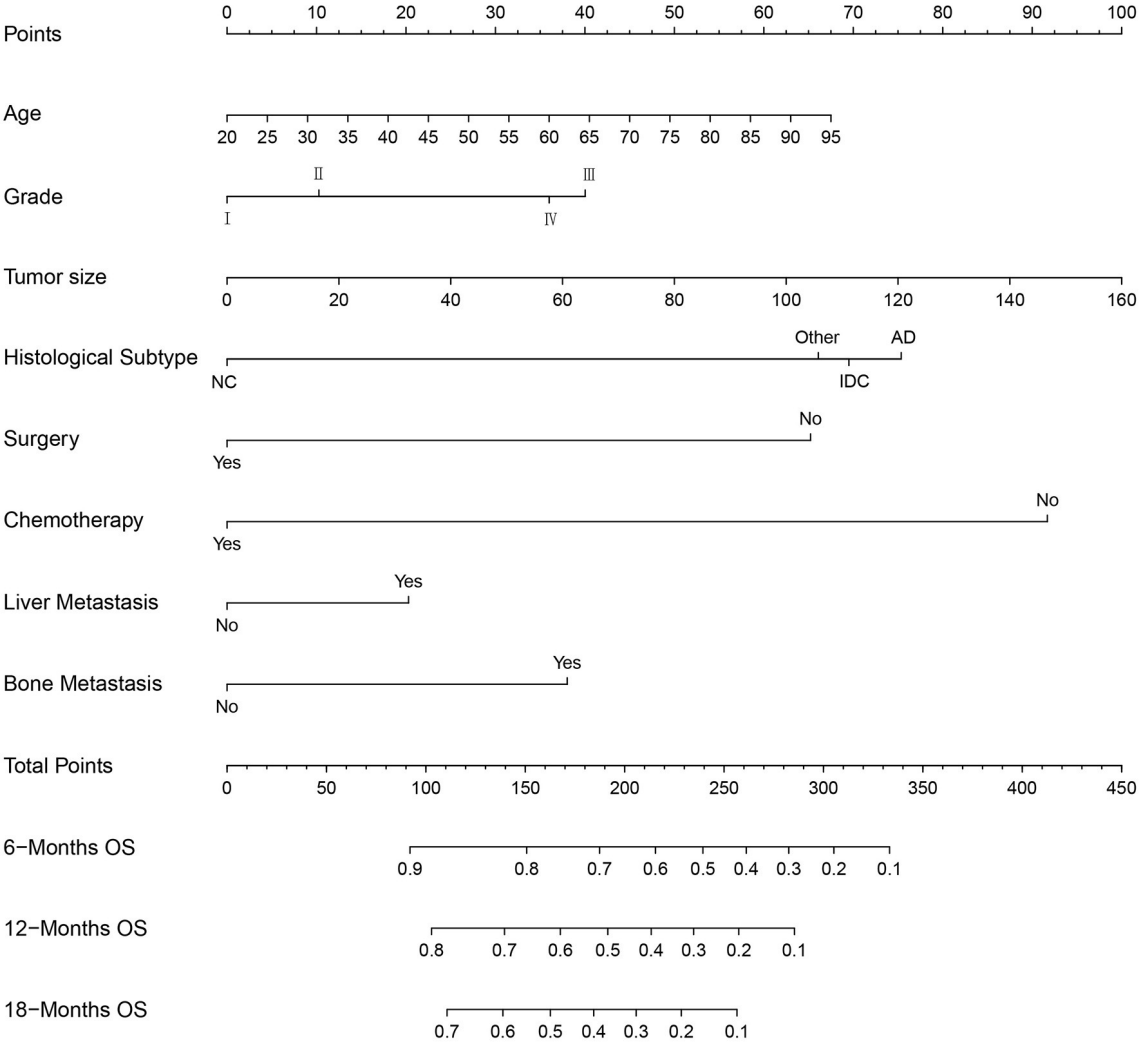
Nomogram for predicting the overall survival of patients with pancreatic cancer presenting with lung metastasis. To use this nomogram, the specific point for each variable of the patient lies on each variable axis. Draw a vertical line upward to determine the point at which each variable accepts; the sum of these points is located on the Total Points axis, and draw a vertical line down to the survival axis to determine the probability of 6-, 12- and 18- months overall survival.

**Figure 7:**
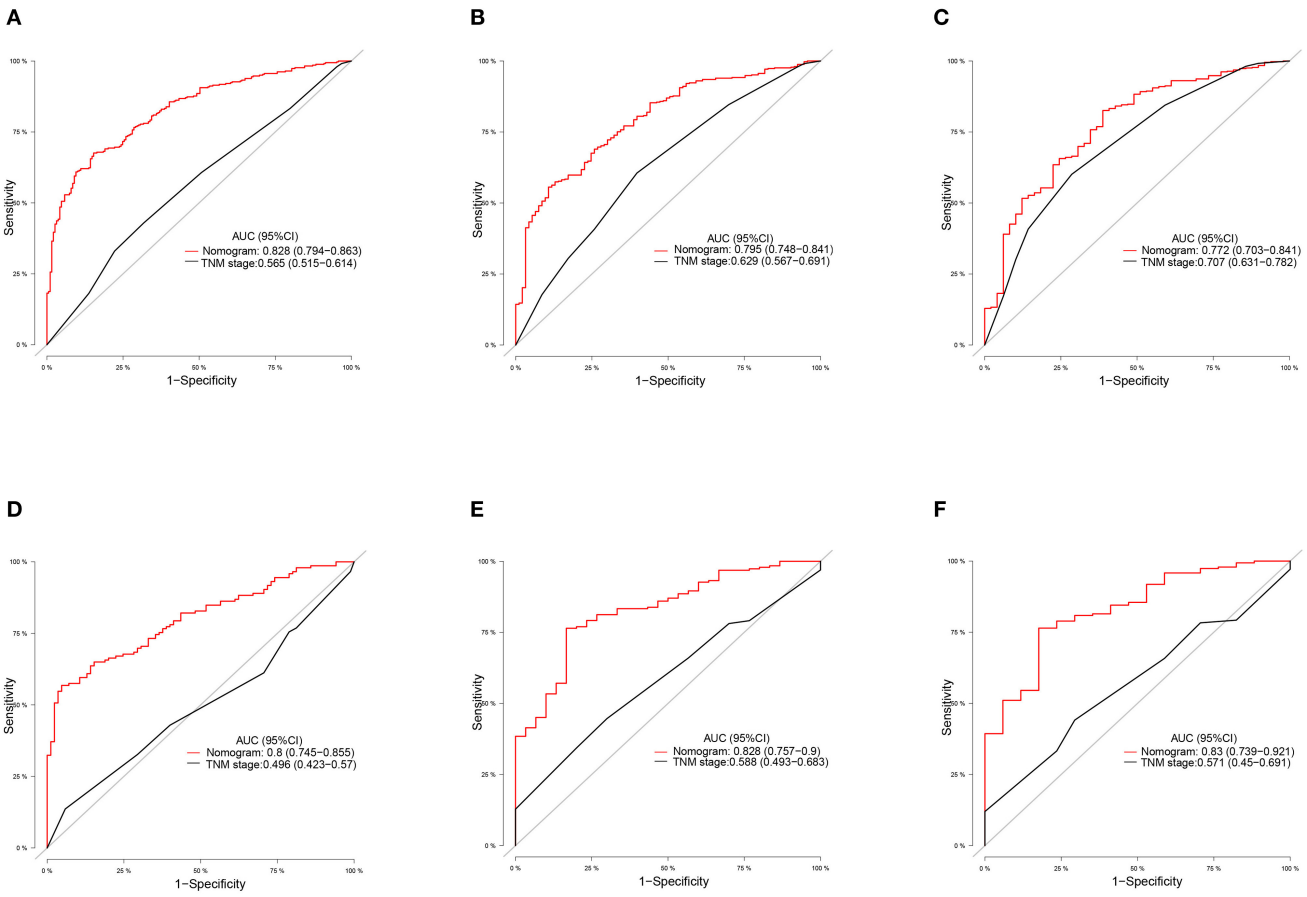
ROC curves of the ability of nomogram and TNM stage to predict 6-, 12- and 18-months overall survival in **(A–C)** training cohort and **(D–F)** validation cohort.

**Figure 8:**
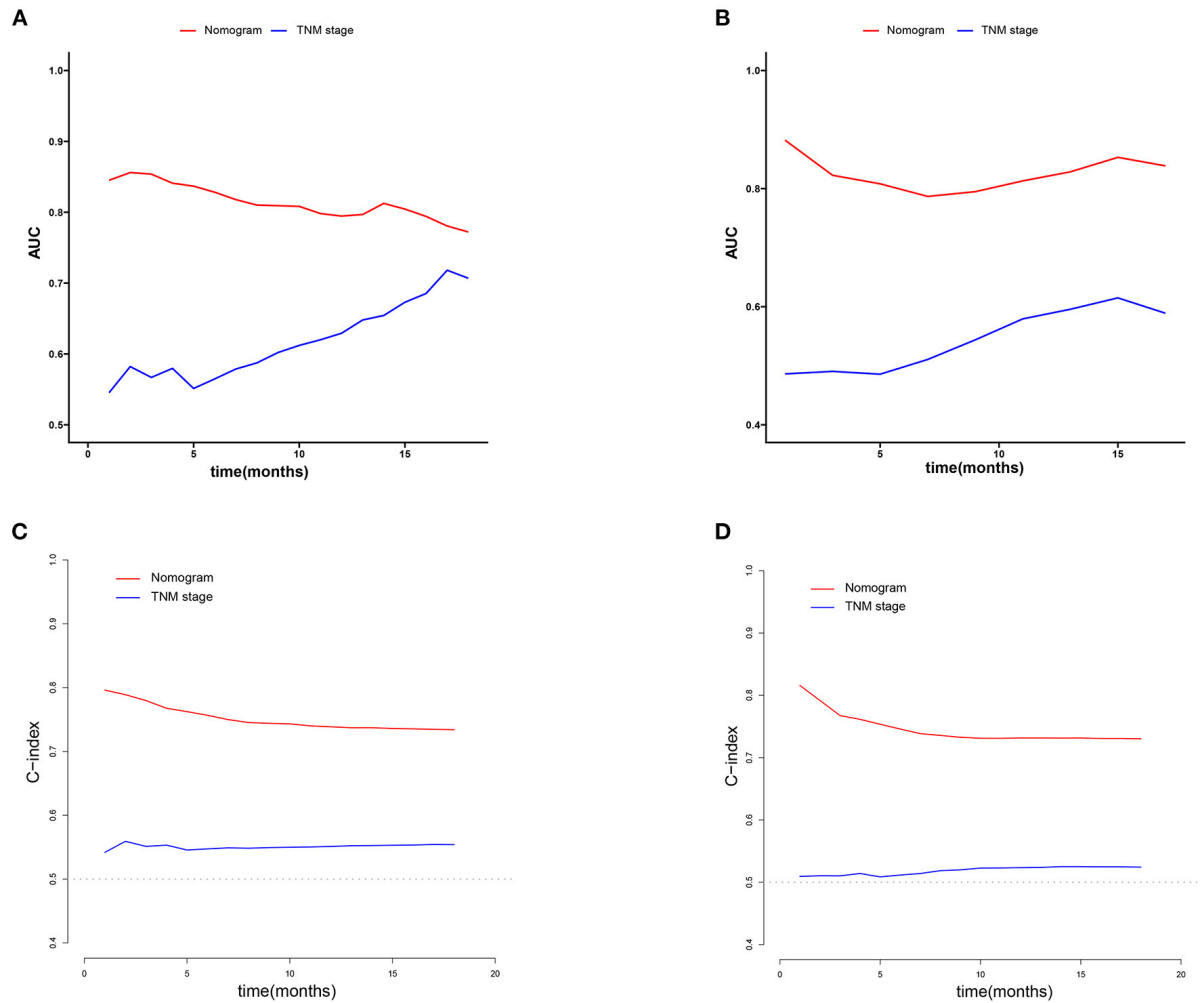
Time-dependent ROC and C-index curves of overall survival. **(A,B)** The time-dependent ROC curves corresponding to 1–15 months in the training cohort and the verification cohort. **(C,D)** The time-dependent C-index curves corresponding to 1–20 months in the training cohort and the verification cohort.

**Figure 9:**
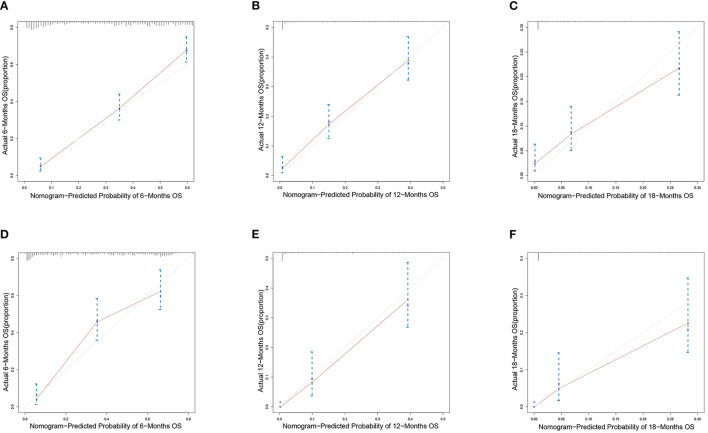
Calibration curves of the nomograms. Calibration curves of 6-, 12- and 18-months overall survival for PCLM patients in **(A–C)** training cohort and **(D–F)** verification cohort.

**Figure 10:**
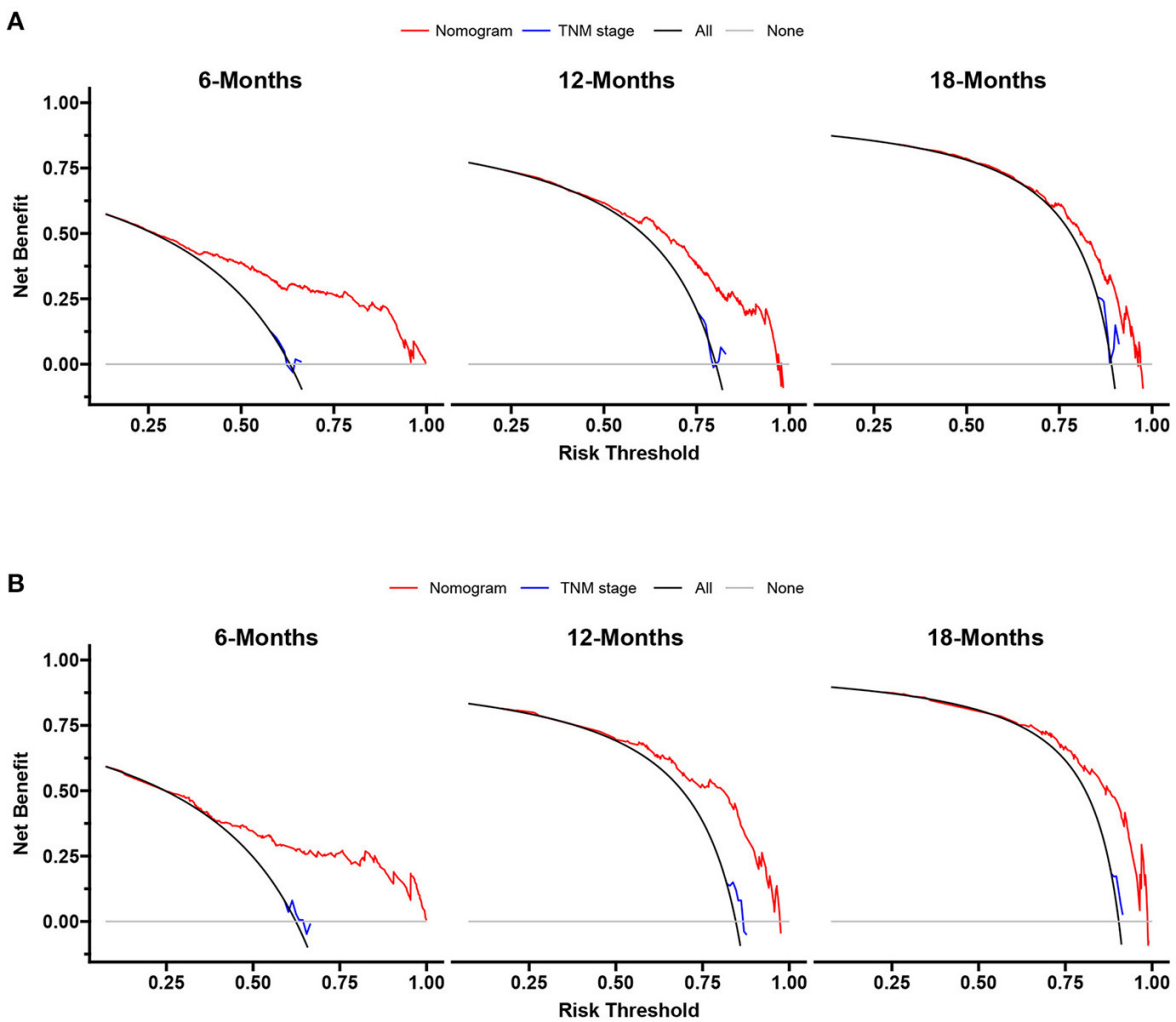
Decision curves of the nomogram and TNM stage for survival prediction of PCLM patients. **(A)** 6-, 12- and 18-months survival benefit in the training cohort. **(B)** 6-, 12- and 18-months survival benefit in the verification cohort.

We constructed prognostic nomograms based on multivariate Cox hazard regression analysis results to demonstrate the impact of independent prognostic factors on OS more intuitively (Figure 6). Furthermore, to make the prognostic nomogram more user-friendly, we have created a free browser-based online calculator available at https://pclmnomogram.shinyapps.io/CoxNomogram/. The AUC values were 0.828 (CI 0.794–0.863), 0.795(CI 0.748–0.841) and 0.772 (CI 0.703–0.841) regarding nomograms predicting 6-, 12-, and 18-months OS in the training cohort (Figures 7A–C). Meanwhile, AUC values were 0.800 (CI 0.745–0.855), 0.828 (CI 0.757–0.900) and 0.830 (CI 0.739–0.921) regarding nomograms predicting 6-, 12-, and 18-months OS in the validation cohort (Figures 7D–F). As shown in Figure 7, whether in predicting 6-, 12-, or 18-months OS, the AUC values of the nomogram outperformed the traditional TNM staging. The time-dependent ROC curves disclosed that AUC value fluctuated at 0.8 from 1 to 18 months in the training cohort. Surprisingly, the fluctuation range of the AUC value of validation cohort was remarkably consistent with that of the training cohort (Figures 8A,B). Then, we used time-dependent C-index curves to compare the effectiveness of the nomogram model, and the results showed that the effect of the nomogram was superior to TNM staging (Figures 8C,D). The calibration curve at 6, 12, and 18 months for OS probabilities of the training cohort was in good agreement with OS predicted by the nomograms to the actual results (Figures 9A–C). The calibration curves for the validation cohort's OS probabilities revealed improved consistency between OS indicated by the nomogram and the actual results (Figures 9D–F). The DCA showed that the clinical value of the nomogram is higher than that of the TNM staging (Figures 10A,B).

## Discussion

PC remains a significant threat to cancer treatment, globally. While it is expected to become the second leading cause of cancer-related death in the next decade, the survival rate of patients with PC has more than doubled due to continuous advances in modern medicine (21). The most common pathological type of PC reported in the literature is pancreatic ductal adenocarcinoma (PDAC) (22, 23). Most PDAC patients have locally advanced or metastatic disease at the time of initial diagnosis, and the incidence of LM is as high as 45% (24). It stated that if medical intervention is not provided on time, the patient's prognosis will be extremely poor. New chemotherapeutic agents prolong survival of patients with PDAC. However, in some special types of PDAC patients, such as patients with end-stage renal disease requiring hemodialysis, they should use the prior-dosing method during chemotherapy (25). Besides, the incidence of PCLM in our study was 4.2%, which could be attributed to our strict inclusion criteria and the inclusion of more pathological types of PC cases. Astonishingly, this is very similar to previous research on lung metastasis in other cancers based on SEER database (26–28). To the best of our knowledge, this is the first population-based study that describes the diagnostic and prognostic factors of PCLM patients. We developed two novel nomograms to predict the diagnosis and prognosis of PCLM patients in our research. Finally, we designed two more user-friendly network-based nomograms, hoping that clinicians will use these resources to formulate individual treatment plans for PCLM patients.

We used descriptive statistics and logistic regression analysis to investigate factors related to PCLM at the time of diagnosis. In addition, we utilized Cox hazard regression analysis and Kaplan-Meier curves to obtain survival estimates. The results of logistic analysis revealed that age at diagnosed, histological subtype, primary site, N stage, surgery, radiotherapy, tumor size, bone metastasis, brain metastasis and liver metastasis were independent factors in the diagnosis of PCLM. Based on Cox hazard regression analysis, we established that age at diagnosis, grade, tumor size, histological subtype, surgery, chemotherapy, liver metastasis and bone metastasis were independent prognostic factors for PCLM patients.

Older persons had a higher proportion of lung metastases in PCLM patients regarding age at diagnosis. This finding was consistent with many studies, which show age as an independent risk factor for distant metastases (29–31). We suspected that it might be due to various changes that have taken place in the body's metabolism and development with age. Children's bodies have not yet fully developed, whereas the elderly gradually age. The children's immune system has not been fully matured, and aging is accompanied by cellular senescence, including changes in homeostasis, protein and nuclear genome instability, all of which may be linked to the occurrence and progression of tumors (32–34). Concerning histological subtype, adenocarcinoma and infiltrating duct carcinoma are more likely to develop lung metastasis. As for the primary site, body of pancreas and tail of pancreas are the main risk factors for metastases. N1 tumors have a higher proportion of LM than N0 tumors in N staging. It was previously reported that the T and N staging has the most significant contribution to metastasis prediction (35). Preceding research has shown that patients with larger metastatic lymph nodes are more likely to develop distant metastatic disease (36). Our analysis showed that surgical treatment could suggestively reduce the risk of LM. This study may provide further evidence for pancreatic cancer patients treated with surgery-first (SF) approaches (37, 38). According to our findings, tumor size affected the occurrence of LM. Tumor oxygenation decreased with tumor volume in the rodent tumor model KHT-C, and hypoxic tumors were more likely to metastasize. These results were consistent with clinical data, indicating that the hypoxic environment due to tumor size changes may be involved in the metastatic ability of human tumors (39, 40). As expected, patients with bone, brain, or liver metastases were more likely to have lung metastasis. CT scanning is typically used to detect lung metastases; however, this imaging technique has apparent shortcomings in detecting early metastatic lesions in the lung (41). Computer-aided detection (CAD) of pulmonary nodules has advanced recently, particularly in detecting small pulmonary nodules. The CAD system can improve sensitivity in diagnosing pulmonary nodules and reduce false-positive rates, particularly in small and isolated nodules (42). To summarize, we strongly advise that high-risk PC patients be screened for lung nodules early and, if necessary, undergo lung biopsy to ensure early diagnosis of lung metastases.

The Cox model is a multivariate semiparametric regression model that is now widely used in clinical research to characterize disease progression in existing cases by revealing the importance of covariates. The proportional hazard model is the most general regression model because it makes no assumptions about the nature or shape of the potential survival distribution. As a result, the Cox proportional hazard regression model is used to evaluate the correlation between the exposure of interest in the observed data and the time outcome of the event (43). The results of a multivariate Cox regression analysis revealed that the higher the degree of grading, the worse the patients' prognosis. This finding, like many others, suggested that histological grading plays a vital role in predicting patient survival (44, 45). Our study disclosed that older patients and large tumor sizes had significantly lower overall survival. A decline in immunity and metabolic capacity with the aging of natural state could cause a worse prognosis; furthermore, as per our findings, patients with adenocarcinoma and infiltrating duct carcinoma also had worse overall survival compared to those with other histological subtypes due to its aggressive metastatic spread (46). In terms of treatment, surgery and chemotherapy positively impacted the overall survival of PCLM patients. Distant metastasis accompanied by liver or bone metastases harmed the prognosis of PCLM patients.

According to Kaplan-Meier curves, surgery and chemotherapy increased the median survival of PCLM patients by 7 and 5.5 months, respectively, compared to unoperated and chemotherapy-free patients. Furthermore, when compared to surgery or chemotherapy alone, surgery combined with chemotherapy increased the median survival of PCLM patients by 11.5 or 10 months. It validates the findings of previous PC studies that surgical resection combined with systemic chemotherapy is currently the only option for long-term survival. Improvements in surgical safety and effectiveness have resulted in a perioperative mortality rate of about 3% and a 5-year survival rate of nearly 30% after resection and adjuvant chemotherapy. Because of advancements in surgical techniques and systemic chemotherapy, indications for resection now include locally advanced tumors (47). However, there is still a high risk of postoperative complications. Unfortunately, we were unable to conduct an in-depth analysis of the survival of PCLM patients with postoperative complications due to a lack of records in the database. Therefore, we are excited to investigate the impact of postoperative complications on the survival of PCLM patients in the prospective follow-up study. There was also a statistically significant difference in median survival between liver metastases and bone metastases (*p* < 0.001; *p* < 0.001). Based on research findings, we found that more extrapulmonary metastases were consistently associated with poor survival, a trend that was consistent with other malignancies (48, 49). As a result, additional metastatic sites were frequently associated with a poor prognosis of malignant tumors.

Of course, there are some limitations in our study. First, it is a retrospective study based on SEER database, that may contain some unavoidable bias. Second, the data recorded in the SEER database is limited, while some clinical factors, critical laboratory and biochemical indicators were unavailable, such as Body Mass Index (BMI), drinking, smoking, tumor biomarkers, blood routine and so on. Third, all analyses are based on the population of the United States, which may not be representative of the people of other counties or regions. Finally, the nomogram we constructed is internally validated against the SEER database, but lacks validation with external data. Thus, it is necessary to further utilize external validation to check the accuracy and reliability of the predictive model. We have collected partial data in the Chinese population and expect to externally validate the predictive model in the near future.

## Conclusion

To the best of our knowledge, this is the first population-based study to diagnose and predict the prognosis of PCLM patients. We analyzed the independent risk factors for diagnosis and independent predictive factors of PCLM patients' prognosis and developed two visual nomograms. We affirmed that these nomograms have excellent accuracy and differentiation using AUC, C-index and calibration curves. DCA showed that these nomograms had good clinical utility. Subsequently, we developed two web-based nomograms to help clinicians make early diagnoses, choose appropriate treatment strategies for PCLM patients, and ultimately maximize the prognostic benefits of these patients.

## Data Availability Statement

The original contributions presented in the study are included in the article/supplementary material, further inquiries can be directed to the corresponding author/s.

## Ethics Statement

The studies involving human participants were reviewed and approved by Ethics Committee of the Zhejiang Provincial People's Hospital. Written informed consent for participation was not required for this study in accordance with the national legislation and the institutional requirements. Written informed consent was not obtained from the individual(s) for the publication of any potentially identifiable images or data included in this article.

## Author Contributions

QB and YK designed the project, reviewed, and edited the manuscript. WZ wrote the manuscript. LJ, XZ, and SZ contributed to literature retrieval. YZ and MG carried out research selection, data extraction, and statistical analysis. WZ and LJ prepared tables and figures. All authors contributed to this article and approved the submitted version.

## Funding

This work was supported by the Key Research and Development Program of Zhejiang Province (2021C03078).

## Conflict of Interest

The authors declare that the research was conducted in the absence of any commercial or financial relationships that could be construed as a potential conflict of interest.

## Publisher's Note

All claims expressed in this article are solely those of the authors and do not necessarily represent those of their affiliated organizations, or those of the publisher, the editors and the reviewers. Any product that may be evaluated in this article, or claim that may be made by its manufacturer, is not guaranteed or endorsed by the publisher.
